# Supplementary material for: Interplay Between Dysregulated Immune System and the Footprints of Blood-Borne miRNAs in Treatment Naive Crohn’s Disease and Ulcerative Colitis Patients
Source: Int J Mol Sci. 2025 Dec 15;26(24):12042. doi: 10.3390/ijms262412042 (PMC12732772; doi:10.3390/ijms262412042)
Supplement: Supplementary file 1 [file ijms-26-12042-s001.zip › Supplementary_Figure S1.pdf]

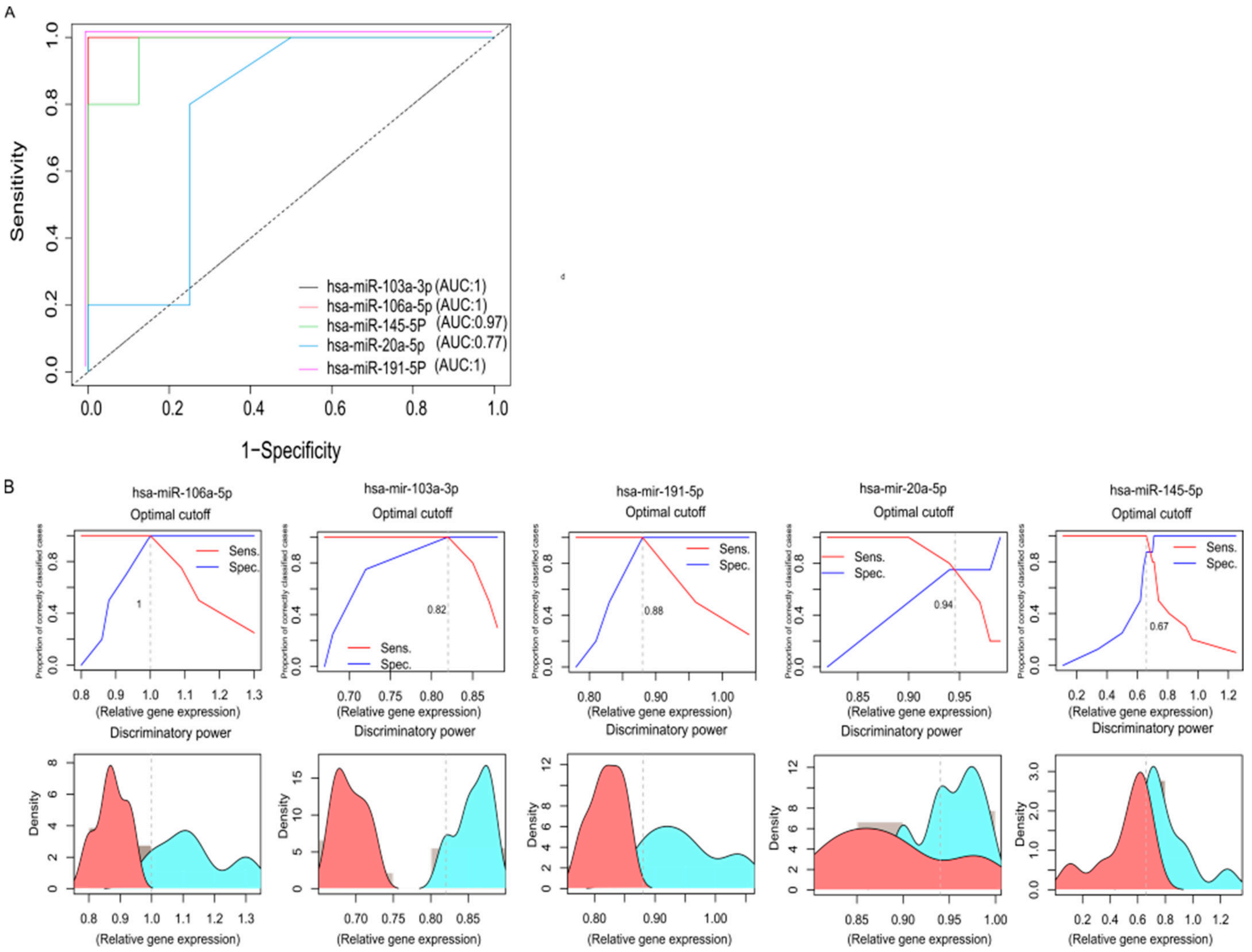


**Supplementary Figure S1:** ROC (Receiver Operating Characteristics) analyses illustrate the diagnostic performance of the 5 microRNAs, having significantly different expression levels between CD and UC groups, at different discriminatory thresholds (A). ROC analyses were performed based on relative gene expression data to assess the diagnostic potential of miRNAs between CD and UC study groups. Line graphs were used to calculate the optimal cut-off points (B). Discriminatory power graphs

represent the distribution of the relative gene expression values in CD and UC groups (D). CD (Crohn's disease), UC (Ulcerative colitis), RPM (read per million).
