# Supplementary material for: Interplay Between Dysregulated Immune System and the Footprints of Blood-Borne miRNAs in Treatment Naive Crohn’s Disease and Ulcerative Colitis Patients
Source: Int J Mol Sci. 2025 Dec 15;26(24):12042. doi: 10.3390/ijms262412042 (PMC12732772; doi:10.3390/ijms262412042)
Supplement: Supplementary file 1 [file ijms-26-12042-s001.zip › Supplementary_Table S1.pdf]

	miRNA	log2 Fold-change	p-value	Differentially expressed
**1**	hsa-miR-10395-3p	21.07762612	0.113831	NO
**2**	hsa-miR-1229-3p	3.152642355	0.377598	NO
**3**	hsa-miR-1238-3p	2.638634236	0.145202	NO
**4**	hsa-miR-1248	20.5620656	0.508235	NO
**5**	hsa-miR-195-3p	21.61571191	0.497924	NO
**6**	hsa-miR-195-5p	-0.874044704	0.160413	NO
**7**	hsa-miR-3173-5p	1.473577737	0.176623	NO
**8**	hsa-miR-378g	-0.29739346	0.742607	NO
**9**	hsa-miR-4433b-5p	1.824464519	0.586084	NO
**10**	hsa-miR-485-3p	3.315299024	0.496674	NO
**11**	hsa-miR-511-5p	3.296872108	0.403833	NO
**12**	hsa-miR-543	3.576357735	0.388439	NO
**13**	hsa-miR-625-5p	1.694738717	0.252549	NO
**14**	hsa-miR-6509-3p	3.727147359	0.500938	NO
**15**	hsa-miR-6833-3p	0.150667568	0.918223	NO
**16**	hsa-miR-6855-5p	2.088044607	0.368669	NO
**17**	hsa-miR-92b-3p	0.796456599	0.370876	NO
**18**	hsa-miR-937-3p	3.607375235	0.048419	UP
**19**	hsa-miR-943-3p	3.208570838	0.162433	NO
**20**	hsa-miR-485-5p	5.74945445	0.460976	NO
**21**	hsa-miR-6803-3p	0.27751172	0.969912	NO
**22**	hsa-miR-431-5p	-0.5215763	0.371103	NO
**23**	hsa-miR-493-5p	3.568636622	0.377532	NO
**24**	hsa-miR-6857-3p	0.796845066	0.383997	NO
**25**	hsa-let-7f-1-3p	0.615239017	0.573395	NO
**26**	hsa-miR-224-5p	4.060526205	0.477935	NO
**27**	hsa-miR-2355-3p	-1.537734753	0.018958	DOWN
**28**	hsa-miR-24-1-5p	0.095297131	0.920627	NO
**29**	hsa-miR-3146	-0.661781517	0.09531	NO
**30**	hsa-miR-3187-3p	-0.901790349	0.086667	NO
**31**	hsa-miR-338-5p	0.45748854	0.671774	NO
**32**	hsa-miR-3614-5p	2.819816346	0.255756	NO
**33**	hsa-miR-433-3p	1.142009697	0.482785	NO
**34**	hsa-miR-4498	1.645803929	0.400466	NO
**35**	hsa-miR-4503	-0.398393876	0.290227	NO
**36**	hsa-miR-4797-3p	0.861650459	0.194146	NO
**37**	hsa-miR-5582-3p	-0.569028578	0.158123	NO
**38**	hsa-miR-6733-5p	0.014657279	0.915476	NO
**39**	hsa-miR-6735-5p	-0.154889609	0.799637	NO
**40**	hsa-miR-6780a-5p	0.113560479	0.851426	NO
**41**	hsa-miR-942-3p	-0.075958584	0.813375	NO
**42**	hsa-let-7b-3p	1.441089122	0.172631	NO
**43**	hsa-miR-127-5p	1.247775011	0.653763	NO
**44**	hsa-miR-130a-5p	-1.807486007	0.081744	NO

**45**	hsa-miR-196a-5p	-0.246438023	0.824858	NO
**46**	hsa-miR-200b-3p	-0.419985808	0.279482	NO
**47**	hsa-miR-26a-2-3p	0.347028358	0.588289	NO
**48**	hsa-miR-3160-3p	-0.256374091	0.578229	NO
**49**	hsa-miR-3934-5p	-0.277036679	0.563144	NO
**50**	hsa-miR-4446-3p	6.304689999	0.502737	NO
**51**	hsa-miR-4474-3p	-1.079023158	0.073927	NO
**52**	hsa-miR-570-3p	-2.071640666	0.000955	DOWN
**53**	hsa-miR-6505-5p	-0.474879212	0.319748	NO
**54**	hsa-miR-6515-5p	0.484371071	0.528546	NO
**55**	hsa-miR-664b-3p	-2.555622734	5e-06	DOWN
**56**	hsa-miR-6741-3p	2.438019639	0.263331	NO
**57**	hsa-miR-6837-3p	0.361372356	0.563402	NO
**58**	hsa-miR-6894-3p	1.562736174	0.298961	NO
**59**	hsa-miR-889-3p	2.04335887	0.443035	NO
**60**	hsa-let-7f-2-3p	-1.201379105	0.015702	DOWN
**61**	hsa-miR-1178-3p	1.635994158	0.297026	NO
**62**	hsa-miR-1277-5p	0.576180324	0.716429	NO
**63**	hsa-miR-1288-3p	1.626868083	0.034416	UP
**64**	hsa-miR-1289-3p	-0.678366365	0.155326	NO
**65**	hsa-miR-136-3p	-0.417454507	0.634883	NO
**66**	hsa-miR-181c-5p	-1.338625946	0.003085	DOWN
**67**	hsa-miR-23b-5p	2.33026797	0.325429	NO
**68**	hsa-miR-23c	-3.328805043	0.000139	DOWN
**69**	hsa-miR-31-5p	-2.325466968	0.000414	DOWN
**70**	hsa-miR-3164-5p	-0.639648594	0.072381	NO
**71**	hsa-miR-3683-5p	-1.677284825	0.008636	DOWN
**72**	hsa-miR-452-5p	-0.475719533	0.548595	NO
**73**	hsa-miR-4690-3p	0.351768447	0.521285	NO
**74**	hsa-miR-4742-5p	0.387352705	0.634668	NO
**75**	hsa-miR-4755-5p	0.69191381	0.316327	NO
**76**	hsa-miR-5000-3p	0.857878351	0.234147	NO
**77**	hsa-miR-6503-3p	-2.263060762	0.000118	DOWN
**78**	hsa-miR-6882-5p	-1.334431168	0.024956	DOWN
**79**	hsa-miR-7855-5p	0.030493505	0.959516	NO
**80**	hsa-miR-874-3p	-2.331450496	2.1e-05	DOWN
**81**	hsa-miR-1273h-3p	2.463376949	0.359947	NO
**82**	hsa-miR-1468-5p	2.604523398	0.009361	UP
**83**	hsa-miR-16-1-3p	-2.477808744	0.001146	DOWN
**84**	hsa-miR-2277-5p	-2.480009654	0.000204	DOWN
**85**	hsa-miR-3138-3p	0.92041803	0.049403	NO
**86**	hsa-miR-3150b-3p	0.196762911	0.714371	NO
**87**	hsa-miR-3163	1.264487015	0.046382	UP
**88**	hsa-miR-3202	-0.207548326	0.86757	NO
**89**	hsa-miR-382-3p	0.021488029	0.890985	NO
**90**	hsa-miR-4661-5p	2.594013082	0.016804	UP

**91**	hsa-miR-4762-3p	-0.152431621	0.796136	NO
**92**	hsa-miR-491-5p	-1.002776971	0.114833	NO
**93**	hsa-miR-5584-5p	-0.67149621	0.764672	NO
**94**	hsa-miR-6815-5p	1.073329266	0.043486	UP
**95**	hsa-miR-1246	-3.000217704	0.401066	NO
**96**	hsa-miR-1285-5p	-1.588114312	0.002143	DOWN
**97**	hsa-miR-188-5p	-1.937175302	0.003837	DOWN
**98**	hsa-miR-3136-5p	-1.481169729	0.000494	DOWN
**99**	hsa-miR-3199-5p	-0.908115011	0.091591	NO
**100**	hsa-miR-375-3p	1.241697058	0.101215	NO
**101**	hsa-miR-411-5p	1.498916939	0.551562	NO
**102**	hsa-miR-4646-5p	-0.12840367	0.958086	NO
**103**	hsa-miR-5001-3p	-1.290976925	0.003703	DOWN
**104**	hsa-miR-5010-5p	0.716359738	0.285706	NO
**105**	hsa-miR-514a-3p	0.100335973	0.855668	NO
**106**	hsa-miR-548az-5p	0.520810095	0.436433	NO
**107**	hsa-miR-627-3p	-1.789755314	0.000277	DOWN
**108**	hsa-miR-6747-3p	2.029450932	0.076955	NO
**109**	hsa-miR-6783-3p	1.229461198	0.084759	NO
**110**	hsa-miR-6818-3p	0.482467826	0.392627	NO
**111**	hsa-miR-106a-3p	-1.618630727	0.001436	DOWN
**112**	hsa-miR-1284-5p	-0.008746237	0.819595	NO
**113**	hsa-miR-145-3p	0.145109462	0.884491	NO
**114**	hsa-miR-184	0.524597214	0.372611	NO
**115**	hsa-miR-3064-5p	0.170916981	0.641244	NO
**116**	hsa-miR-3133	-0.048435415	0.941719	NO
**117**	hsa-miR-3179-3p	-0.051591387	0.725616	NO
**118**	hsa-miR-32-3p	0.342639048	0.769571	NO
**119**	hsa-miR-374b-3p	-0.892597871	0.02359	NO
**120**	hsa-miR-499a-5p	0.533240201	0.293838	NO
**121**	hsa-miR-5189-3p	0.459194255	0.497695	NO
**122**	hsa-miR-548q	-0.367279717	0.336684	NO
**123**	hsa-miR-597-3p	-0.415987795	0.365627	NO
**124**	hsa-miR-636-3p	-0.988051078	0.045262	NO
**125**	hsa-miR-6750-5p	-0.437158151	0.264975	NO
**126**	hsa-miR-10b-5p	3.477750153	0.370438	NO
**127**	hsa-miR-1908-5p	3.225703912	0.322071	NO
**128**	hsa-miR-3120-3p	-0.952886461	0.164385	NO
**129**	hsa-miR-3157-3p	-0.645224355	0.063172	NO
**130**	hsa-miR-3613-3p	-2.126548024	0.001395	DOWN
**131**	hsa-miR-374c-5p	-1.921877482	0.361027	NO
**132**	hsa-miR-3912-3p	-0.926121027	0.010382	NO
**133**	hsa-miR-409-5p	0.537542056	0.698052	NO
**134**	hsa-miR-4286	-2.261171601	0.003578	DOWN
**135**	hsa-miR-4672-3p	-0.213662883	0.616834	NO
**136**	hsa-miR-4678-5p	-0.348552009	0.349879	NO

**137**	hsa-miR-501-5p	-1.739660343	0.000998	DOWN
**138**	hsa-miR-628-5p	-0.797226042	0.03026	NO
**139**	hsa-miR-7854-3p	-0.332175643	0.884236	NO
**140**	hsa-miR-1306-5p	-0.490361727	0.143491	NO
**141**	hsa-miR-139-3p	4.483705116	0.494796	NO
**142**	hsa-miR-30b-3p	0.563358083	0.381151	NO
**143**	hsa-miR-323b-3p	1.327828258	0.388086	NO
**144**	hsa-miR-584-3p	-1.539220643	0.001346	DOWN
**145**	hsa-miR-618-5p	-0.786352809	0.180022	NO
**146**	hsa-miR-628-3p	-0.087773786	0.568342	NO
**147**	hsa-miR-6511b-3p	-1.472178161	0.001053	DOWN
**148**	hsa-miR-654-3p	1.855671514	0.503682	NO
**149**	hsa-miR-766-3p	-2.230643963	6.1e-05	DOWN
**150**	hsa-let-7g-3p	-1.54846934	0.006771	DOWN
**151**	hsa-miR-101-5p	-2.280366341	3e-06	DOWN
**152**	hsa-miR-190b-5p	-1.506152578	4.8e-05	DOWN
**153**	hsa-miR-301a-5p	-1.01576557	0.078644	NO
**154**	hsa-miR-30c-1-3p	0.230294005	0.797343	NO
**155**	hsa-miR-3176-3p	0.225466148	0.595749	NO
**156**	hsa-miR-505-5p	-0.002446669	0.80416	NO
**157**	hsa-miR-5189-5p	1.435537893	0.276293	NO
**158**	hsa-miR-548ac	-0.983279867	0.010888	NO
**159**	hsa-miR-610	-0.648823472	0.109385	NO
**160**	hsa-miR-654-5p	3.684224086	0.44124	NO
**161**	hsa-miR-660-3p	-3.026210206	3.2e-05	DOWN
**162**	hsa-miR-6868-3p	0.233578211	0.714008	NO
**163**	hsa-miR-6877-5p	-0.417318274	0.453959	NO
**164**	hsa-miR-122-5p	1.5805361	0.4112	NO
**165**	hsa-miR-132-5p	0.360278125	0.551728	NO
**166**	hsa-miR-3177-3p	0.638102016	0.430512	NO
**167**	hsa-miR-4637	-0.715050796	0.085101	NO
**168**	hsa-miR-4742-3p	-0.044902469	0.744082	NO
**169**	hsa-miR-494-3p	-1.269490257	0.007916	DOWN
**170**	hsa-miR-6513-3p	-0.538305062	0.075689	NO
**171**	hsa-miR-99b-3p	1.772407834	0.556936	NO
**172**	hsa-miR-579-5p	-1.352757209	0.015747	DOWN
**173**	hsa-miR-6511a-3p	0.286185282	0.840444	NO
**174**	hsa-miR-1273c	-1.229517733	0.007147	DOWN
**175**	hsa-miR-362-3p	-2.833534681	1e-06	DOWN
**176**	hsa-miR-4668-5p	-1.815276473	0.000582	DOWN
**177**	hsa-miR-548u-3p	-1.528672062	0.000903	DOWN
**178**	hsa-miR-641	-0.380790613	0.565714	NO
**179**	hsa-miR-3140-3p	-0.194730299	0.545943	NO
**180**	hsa-miR-3691-5p	0.391121317	0.339801	NO
**181**	hsa-miR-6806-3p	0.34385768	0.502412	NO
**182**	hsa-miR-939-5p	0.114978243	0.755605	NO

**183**	hsa-miR-150-3p	0.409044506	0.630897	NO
**184**	hsa-miR-335-3p	1.291924715	0.469159	NO
**185**	hsa-miR-4521	-1.771406504	1.3e-05	DOWN
**186**	hsa-miR-548p	-0.794990752	0.034588	NO
**187**	hsa-miR-671-3p	4.590791485	0.347341	NO
**188**	hsa-miR-7-1-3p	-1.108643488	0.001956	DOWN
**189**	hsa-miR-3128	-0.262897753	0.587562	NO
**190**	hsa-miR-6514-5p	0.822666669	0.053861	NO
**191**	hsa-miR-219a-1-3p	0.81904616	0.050945	NO
**192**	hsa-miR-3605-3p	2.89969375	0.057303	NO
**193**	hsa-miR-4473-3p	0.308927054	0.583045	NO
**194**	hsa-miR-550a-3p	-2.775781325	1e-06	DOWN
**195**	hsa-miR-1306-3p	-0.256078856	0.341523	NO
**196**	hsa-miR-3909-3p	-0.400401747	0.238755	NO
**197**	hsa-miR-580-3p	-1.745846008	0.003165	DOWN
**198**	hsa-miR-6852-5p	2.008989783	0.497577	NO
**199**	hsa-miR-6866-5p	0.705767661	0.091276	NO
**200**	hsa-miR-3605-5p	0.132694651	0.617054	NO
**201**	hsa-miR-6884-5p	-0.73748356	0.344157	NO
**202**	hsa-miR-495-3p	1.112049716	0.583122	NO
**203**	hsa-miR-5009-5p	1.102441231	0.076404	NO
**204**	hsa-miR-6734-5p	-0.348042595	0.285282	NO
**205**	hsa-miR-92b-5p	2.3667996	0.098733	NO
**206**	hsa-miR-1303-3p	0.221162254	0.769544	NO
**207**	hsa-miR-183-3p	-2.398948716	1e-06	DOWN
**208**	hsa-miR-363-5p	0.991009014	0.053379	NO
**209**	hsa-miR-378a-5p	-1.477522211	0.000243	DOWN
**210**	hsa-miR-382-5p	1.024289696	0.586951	NO
**211**	hsa-miR-432-5p	3.690326524	0.502681	NO
**212**	hsa-miR-581-5p	-0.065589138	0.787006	NO
**213**	hsa-miR-3200-5p	-2.17512487	1.3e-05	DOWN
**214**	hsa-miR-5695-3p	-1.371530053	0.000101	DOWN
**215**	hsa-let-7a-3p	2.475078304	0.027625	UP
**216**	hsa-miR-10527-5p	-0.187760143	0.556403	NO
**217**	hsa-miR-132-3p	-1.148120564	0.002423	DOWN
**218**	hsa-miR-3135a	-1.37609832	0.002278	DOWN
**219**	hsa-miR-486-3p	7.73427246	0.020519	UP
**220**	hsa-miR-30a-3p	2.550629553	0.26272	NO
**221**	hsa-miR-4685-3p	0.058391199	0.992926	NO
**222**	hsa-miR-145-5p	-2.298759338	0.000397	DOWN
**223**	hsa-miR-3143	-1.227547395	0.000225	DOWN
**224**	hsa-miR-4669	-0.272459757	0.864827	NO
**225**	hsa-miR-505-3p	-1.814976489	3.8e-05	DOWN
**226**	hsa-miR-664a-3p	-0.950449337	0.017424	NO
**227**	hsa-miR-147b-3p	-0.01491855	0.956773	NO
**228**	hsa-miR-1292-5p	0.398740611	0.344203	NO

**229**	hsa-miR-424-5p	-2.472255795	3.5e-05	DOWN
**230**	hsa-miR-1224-5p	-0.680390379	0.294237	NO
**231**	hsa-miR-18b-5p	-1.91840936	2e-05	DOWN
**232**	hsa-miR-30d-3p	-0.72273979	0.002284	NO
**233**	hsa-miR-16-2-3p	3.309681285	0.009431	UP
**234**	hsa-miR-25-5p	0.605925927	0.487255	NO
**235**	hsa-miR-130b-5p	1.548852708	0.236162	NO
**236**	hsa-miR-193a-5p	0.064530259	0.899994	NO
**237**	hsa-miR-3074-5p	-0.597826497	0.038395	NO
**238**	hsa-miR-4511-5p	-1.344820461	0.000291	DOWN
**239**	hsa-miR-6501-5p	-1.196897338	0.031409	DOWN
**240**	hsa-miR-6859-5p	-0.010262407	0.907959	NO
**241**	hsa-miR-146b-3p	-0.04028751	0.848391	NO
**242**	hsa-miR-10399-5p	-0.036566376	0.78209	NO
**243**	hsa-miR-29b-2-5p	-2.339740874	1e-06	DOWN
**244**	hsa-miR-625-3p	0.085284089	0.946987	NO
**245**	hsa-miR-574-3p	-0.700275603	0.031783	NO
**246**	hsa-miR-200c-3p	-0.110495945	0.473687	NO
**247**	hsa-miR-574-5p	0.327428962	0.759583	NO
**248**	hsa-miR-3928-3p	-0.027908947	0.849087	NO
**249**	hsa-miR-26b-3p	-1.528432263	0.000985	DOWN
**250**	hsa-miR-181d-5p	-0.237087601	0.747567	NO
**251**	hsa-miR-9-5p	-0.302973215	0.305351	NO
**252**	hsa-miR-1270-5p	0.14494282	0.956556	NO
**253**	hsa-miR-21-3p	-0.985114028	0.029943	NO
**254**	hsa-miR-454-5p	-0.426019616	0.18565	NO
**255**	hsa-miR-11400	2.947437863	0.461394	NO
**256**	hsa-miR-493-3p	1.949798521	0.396583	NO
**257**	hsa-miR-450b-5p	-0.020470548	0.739497	NO
**258**	hsa-miR-27a-5p	0.945900993	0.371728	NO
**259**	hsa-miR-582-3p	2.057723128	0.089201	NO
**260**	hsa-miR-24-2-5p	-0.582618949	0.09516	NO
**261**	hsa-miR-877-5p	-0.24448394	0.42997	NO
**262**	hsa-miR-1307-5p	-1.375651514	0.001638	DOWN
**263**	hsa-miR-139-5p	1.188397316	0.440345	NO
**264**	hsa-miR-190a-5p	-2.17917875	0.000635	DOWN
**265**	hsa-miR-624-5p	-1.615661611	2.1e-05	DOWN
**266**	hsa-miR-331-5p	-0.87618984	0.021648	NO
**267**	hsa-miR-181a-3p	-1.109206708	0.010118	DOWN
**268**	hsa-miR-197-3p	0.097549318	0.932221	NO
**269**	hsa-miR-340-3p	1.618188231	0.07854	NO
**270**	hsa-miR-342-5p	1.149316337	0.060912	NO
**271**	hsa-miR-330-3p	-0.193426264	0.534365	NO
**272**	hsa-miR-1287-5p	-1.469292865	0.000536	DOWN
**273**	hsa-miR-4746-5p	2.233756403	0.001785	UP
**274**	hsa-let-7i-3p	-1.617233272	0.00125	DOWN

**275**	hsa-miR-28-5p	-0.256500113	0.581559	NO
**276**	hsa-miR-659-5p	0.839677525	0.062631	NO
**277**	hsa-miR-379-5p	1.527499396	0.411878	NO
**278**	hsa-miR-221-5p	-0.944619766	0.000636	NO
**279**	hsa-miR-651-5p	-0.627420732	0.098408	NO
**280**	hsa-miR-542-3p	-0.883037597	0.053052	NO
**281**	hsa-miR-4677-3p	0.047659613	0.971246	NO
**282**	hsa-miR-3200-3p	-2.222544823	5e-06	DOWN
**283**	hsa-miR-424-3p	0.645593394	0.168649	NO
**284**	hsa-miR-326-3p	-2.19013224	0.001207	DOWN
**285**	hsa-miR-1301-3p	1.678294904	0.465322	NO
**286**	hsa-miR-29c-3p	-1.658152298	0.001063	DOWN
**287**	hsa-miR-1278	0.367471779	0.423503	NO
**288**	hsa-miR-125a-5p	0.88792469	0.650467	NO
**289**	hsa-miR-942-5p	0.213083135	0.799496	NO
**290**	hsa-miR-17-3p	-1.171124353	0.000923	DOWN
**291**	hsa-miR-1976	0.05945825	0.971303	NO
**292**	hsa-miR-6842-3p	1.573414094	0.009744	UP
**293**	hsa-miR-4732-3p	0.973658438	0.127859	NO
**294**	hsa-miR-11401	1.265795808	0.141504	NO
**295**	hsa-miR-29c-5p	-1.008606658	0.001242	DOWN
**296**	hsa-miR-335-5p	-1.033656112	0.016801	DOWN
**297**	hsa-miR-532-3p	-2.20645227	0.000169	DOWN
**298**	hsa-miR-93-3p	-2.1774932	1e-06	DOWN
**299**	hsa-miR-125b-5p	-0.941759413	0.005101	NO
**300**	hsa-miR-324-3p	-1.959047957	6.2e-05	DOWN
**301**	hsa-miR-598-3p	-0.612874162	0.042419	NO
**302**	hsa-miR-361-5p	-1.179956172	0.001051	DOWN
**303**	hsa-miR-664a-5p	0.480351725	0.143589	NO
**304**	hsa-miR-378i	-0.944500818	0.059207	NO
**305**	hsa-miR-330-5p	-0.315392681	0.311174	NO
**306**	hsa-miR-4326	-0.193740957	0.79318	NO
**307**	hsa-miR-199b-5p	-1.100615068	0.010035	DOWN
**308**	hsa-miR-18a-3p	-0.731014456	0.005464	NO
**309**	hsa-miR-548e-3p	-0.831825557	0.004821	NO
**310**	hsa-miR-1294-5p	0.496791744	0.231038	NO
**311**	hsa-miR-362-5p	-2.020654319	0.000114	DOWN
**312**	hsa-miR-339-5p	-1.755677712	0.000185	DOWN
**313**	hsa-miR-548o-3p	0.375396067	0.432426	NO
**314**	hsa-miR-1-3p	0.232407224	0.879086	NO
**315**	hsa-miR-134-5p	1.158656262	0.547663	NO
**316**	hsa-miR-148a-5p	-0.50750337	0.145755	NO
**317**	hsa-miR-409-3p	3.68041662	0.413381	NO
**318**	hsa-miR-4504-3p	0.14177011	0.776867	NO
**319**	hsa-miR-5010-3p	-0.123682448	0.67636	NO
**320**	hsa-miR-1285-3p	-0.423668182	0.039906	NO

**321**	hsa-miR-22-5p	0.036512444	0.888697	NO
**322**	hsa-miR-3913-5p	0.804891564	0.025378	NO
**323**	hsa-miR-328-3p	0.735557884	0.453519	NO
**324**	hsa-miR-769-5p	-0.119183086	0.785142	NO
**325**	hsa-miR-3688-3p	-1.092393686	0.00279	DOWN
**326**	hsa-miR-196b-5p	-0.64308045	0.003875	NO
**327**	hsa-miR-2110	1.337572009	0.064972	NO
**328**	hsa-miR-548bc	-0.457856572	0.134938	NO
**329**	hsa-miR-127-3p	-0.808464763	0.124366	NO
**330**	hsa-miR-140-5p	-0.642902546	0.00656	NO
**331**	hsa-miR-99b-5p	3.038612191	0.435345	NO
**332**	hsa-miR-32-5p	-0.812030967	0.021901	NO
**333**	hsa-let-7d-3p	3.851105172	0.068405	NO
**334**	hsa-miR-1843	1.332839509	0.012133	UP
**335**	hsa-miR-10399-3p	0.577843134	0.041595	NO
**336**	hsa-miR-370-3p	2.112341885	0.386909	NO
**337**	hsa-miR-421	-1.397969739	2e-05	DOWN
**338**	hsa-miR-342-3p	-2.11707607	1e-06	DOWN
**339**	hsa-miR-324-5p	-1.933814158	7e-06	DOWN
**340**	hsa-miR-331-3p	-2.229337702	0.000306	DOWN
**341**	hsa-miR-381-3p	1.461484606	0.435583	NO
**342**	hsa-miR-18a-5p	-1.667410797	0.000146	DOWN
**343**	hsa-miR-4732-5p	-0.75111699	0.011732	NO
**344**	hsa-miR-155-5p	-0.249012857	0.203222	NO
**345**	hsa-miR-576-5p	-0.655596819	0.010808	NO
**346**	hsa-miR-130b-3p	-1.452627741	0.001242	DOWN
**347**	hsa-miR-7976	-0.903277057	0.000256	NO
**348**	hsa-miR-151a-5p/151b	-0.468981375	0.203596	NO
**349**	hsa-miR-3613-5p	-1.218663911	0.000591	DOWN
**350**	hsa-miR-10a-5p	2.578716539	0.293925	NO
**351**	hsa-miR-425-3p	-1.20565328	4.2e-05	DOWN
**352**	hsa-miR-7706	1.244806671	0.014542	UP
**353**	hsa-miR-15b-3p	-0.618874817	0.080693	NO
**354**	hsa-miR-345-5p	-2.081574715	0.000282	DOWN
**355**	hsa-miR-29b-3p	-1.443298037	2.2e-05	DOWN
**356**	hsa-miR-19a-3p	-0.920200634	0.005024	NO
**357**	hsa-miR-4508	0.10432624	0.825072	NO
**358**	hsa-miR-126-5p	-1.202618556	0.000621	DOWN
**359**	hsa-miR-148b-5p	-1.628928758	1.6e-05	DOWN
**360**	hsa-miR-374a-3p	0.820279976	0.166364	NO
**361**	hsa-miR-660-5p	-1.816295451	1.4e-05	DOWN
**362**	hsa-miR-130a-3p	-1.43988843	0.001256	DOWN
**363**	hsa-miR-181a-2-3p	1.116822686	0.013295	UP
**364**	hsa-miR-199a-5p	0.102933583	0.904447	NO
**365**	hsa-miR-223-5p	1.23065603	0.113026	NO
**366**	hsa-miR-210-3p	-1.346127122	0.000389	DOWN

**367**	hsa-miR-99a-5p	0.216837835	0.69381	NO
**368**	hsa-miR-181b-5p	-0.530807122	0.009408	NO
**369**	hsa-miR-1180-3p	0.319189579	0.534475	NO
**370**	hsa-miR-30a-5p	-0.138778964	0.825671	NO
**371**	hsa-miR-589-5p	0.125707389	0.787357	NO
**372**	hsa-miR-744-5p	2.814293079	0.455815	NO
**373**	hsa-miR-374b-5p	-0.711918321	0.07496	NO
**374**	hsa-miR-185-3p	0.365879573	0.243643	NO
**375**	hsa-miR-503-5p	-0.26488152	0.215949	NO
**376**	hsa-miR-29a-3p	-1.202919971	0.002369	DOWN
**377**	hsa-let-7e-5p	0.223685314	0.87998	NO
**378**	hsa-miR-1299	0.290337107	0.863467	NO
**379**	hsa-miR-501-3p	0.039234494	0.810189	NO
**380**	hsa-miR-28-3p	1.469242863	0.389201	NO
**381**	hsa-miR-150-5p	-1.380035248	0.000299	DOWN
**382**	hsa-miR-96-5p	-1.582074326	0.000135	DOWN
**383**	hsa-miR-152-3p	-0.540392183	0.103905	NO
**384**	hsa-miR-1307-3p	0.886774388	0.46104	NO
**385**	hsa-miR-222-3p	1.412336046	0.515907	NO
**386**	hsa-miR-374a-5p	-1.235183633	0.001001	DOWN
**387**	hsa-miR-15a-5p	-1.88111667	0.000225	DOWN
**388**	hsa-miR-454-3p	-1.735343121	1e-06	DOWN
**389**	hsa-miR-100-5p	0.384066445	0.624503	NO
**390**	hsa-miR-500a-3p/502-3p	-0.977751572	0.000241	NO
**391**	hsa-miR-584-5p	1.100386319	0.206325	NO
**392**	hsa-miR-19b-3p	-1.608504988	1.5e-05	DOWN
**393**	hsa-miR-484	-0.712544726	0.000516	NO
**394**	hsa-miR-340-5p	-0.523870416	0.050808	NO
**395**	hsa-miR-23a-3p/23b-3p	-1.109058949	0.000229	DOWN
**396**	hsa-miR-98-5p	-0.02197363	0.458808	NO
**397**	hsa-miR-223-3p	-1.43935187	0.00051	DOWN
**398**	hsa-miR-24-3p	-0.200017796	0.210035	NO
**399**	hsa-miR-361-3p	-0.367111093	0.101042	NO
**400**	hsa-miR-30e-3p	1.750009104	0.003427	UP
**401**	hsa-miR-576-3p	-1.077383354	0.007961	DOWN
**402**	hsa-miR-221-3p	-0.958311795	0.001468	NO
**403**	hsa-miR-20b-5p	-1.409982753	0.000102	DOWN
**404**	hsa-miR-143-3p	1.33744572	0.303673	NO
**405**	hsa-miR-142-3p	-1.35391368	0.001663	DOWN
**406**	hsa-miR-128-3p	1.677787517	0.021445	UP
**407**	hsa-miR-941	0.792621549	0.276221	NO
**408**	hsa-miR-652-3p	-1.153937711	2.6e-05	DOWN
**409**	hsa-miR-194-5p	-0.642321923	0.048978	NO
**410**	hsa-miR-3158-3p	0.727923709	0.041453	NO
**411**	hsa-miR-106b-5p	-1.695028749	1.6e-05	DOWN
**412**	hsa-miR-3615-3p	0.86194614	0.098951	NO

**413**	hsa-miR-30b-5p/30c-5p	-0.674957412	0.00082	NO
**414**	hsa-miR-423-3p	0.909454221	0.082106	NO
**415**	hsa-miR-15b-5p	-1.371665811	4.6e-05	DOWN
**416**	hsa-miR-144-5p	-0.760534303	0.002425	NO
**417**	hsa-miR-146b-5p	-0.284935747	0.075857	NO
**418**	hsa-miR-629-5p	0.184009428	0.850482	NO
**419**	hsa-miR-27a-3p/27b-3p	-0.147156409	0.110093	NO
**420**	hsa-miR-146a-5p	-1.101024761	0.000914	DOWN
**421**	hsa-miR-425-5p	-1.476171035	1e-06	DOWN
**422**	hsa-miR-26b-5p	0.258333008	0.793714	NO
**423**	hsa-miR-144-3p	-1.918662459	1.4e-05	DOWN
**424**	hsa-let-7d-5p	-0.154947705	0.139141	NO
**425**	hsa-miR-148b-3p	0.031058682	0.69493	NO
**426**	hsa-miR-532-5p	0.235248005	0.641537	NO
**427**	hsa-miR-181a-5p	-1.113374185	0.000941	DOWN
**428**	hsa-miR-192-5p/215-5p	-0.14742161	0.262431	NO
**429**	hsa-miR-183-5p	1.288689296	0.051138	NO
**430**	hsa-miR-20a-5p	-1.304317757	0.000721	DOWN
**431**	hsa-miR-106a-5p/17-5p	-1.500810675	7.8e-05	DOWN
**432**	hsa-miR-22-3p	-0.850501421	0.000843	NO
**433**	hsa-miR-423-5p	1.866517197	0.120169	NO
**434**	hsa-miR-30e-5p	-1.366638255	4.7e-05	DOWN
**435**	hsa-miR-378a-3p/378c/378d/378e	-0.056618698	0.518125	NO
**436**	hsa-miR-93-5p	-1.536895359	1.5e-05	DOWN
**437**	hsa-miR-186-5p	-0.943747082	4.9e-05	NO
**438**	hsa-miR-16-5p	-1.571247287	1.9e-05	DOWN
**439**	hsa-miR-142-5p	-0.484886323	0.011685	NO
**440**	hsa-miR-182-5p	0.616875135	0.1379	NO
**441**	hsa-miR-151a-3p	2.291694732	0.01835	UP
**442**	hsa-miR-106b-3p	-0.07326901	0.299884	NO
**443**	hsa-miR-320a-3p/320b/320c/320d/320e	0.169223167	0.984804	NO
**444**	hsa-miR-191-5p	-1.028145048	0.000357	DOWN
**445**	hsa-miR-363-3p	-0.487394343	0.00281	NO
**446**	hsa-miR-126-3p	-0.13890861	0.285285	NO
**447**	hsa-miR-30d-5p	-0.146875639	0.092333	NO
**448**	hsa-miR-26a-5p	0.618679425	0.144724	NO
**449**	hsa-miR-21-5p	0.006395422	0.839414	NO
**450**	hsa-let-7b-5p	0.606420603	0.147723	NO
**451**	hsa-miR-7-5p	-0.495884504	0.039115	NO
**452**	hsa-miR-103a-3p/107	-1.09897114	0.001216	DOWN
**453**	hsa-let-7a-5p/7c-5p	1.129035715	0.00389	UP
**454**	hsa-miR-148a-3p	2.186499874	0.00144	UP
**455**	hsa-miR-140-3p	-0.243006899	0.324652	NO
**456**	hsa-miR-101-3p	-0.247627664	0.189143	NO
**457**	hsa-miR-92a-3p	1.24046056	0.019914	UP
**458**	hsa-miR-25-3p	0.213116258	0.751309	NO

**459**	hsa-miR-185-5p	-0.884085024	0.00579	NO
**460**	hsa-let-7f-5p	0.815553865	0.040433	NO
**461**	hsa-let-7g-5p	-0.117658141	0.19084	NO
**462**	hsa-miR-486-5p	1.220613907	0.035887	UP
**463**	hsa-let-7i-5p	0.053367153	0.56787	NO
**464**	hsa-miR-451a	-0.743708724	0.004397	NO

Supplementary Table S1: Table represents the 464 miRNAs log2 Fold-change values between inflammatory bowel disease (IBD) and Control groups. P-values calculated with Kruskal-Wallis non-parametric test. P-values were adjusted according to Dunn test.
