# Supplementary material for: Interplay Between Dysregulated Immune System and the Footprints of Blood-Borne miRNAs in Treatment Naive Crohn’s Disease and Ulcerative Colitis Patients
Source: Int J Mol Sci. 2025 Dec 15;26(24):12042. doi: 10.3390/ijms262412042 (PMC12732772; doi:10.3390/ijms262412042)
Supplement: Supplementary file 1 [file ijms-26-12042-s001.zip › Supplementary_Table S3.pdf]

miRNAs	AUC	SE AUC	Lower Limit	Upper Limit	z score	p-value	expression
hsa-miR-570-3p	0.83333	0.08575	-0.0014	0.33473	-3.8873	1.00E-04	down
hsa-miR-664b-3p	0.90741	0.05813	-0.02134	0.20652	-7.00874	0	down
hsa-let-7f-2-3p	0.80247	0.08506	0.03081	0.36425	-3.5558	0.00038	down
hsa-miR-1288-3p	0.80864	0.08557	0.64094	0.97635	3.60706	0.00031	up
hsa-miR-181c-5p	0.85185	0.08318	-0.01489	0.31118	-4.22991	2.00E-05	down
hsa-miR-23c	0.96296	0.03144	-0.02458	0.09866	-14.7254	0	down
hsa-miR-31-5p	0.82099	0.09862	-0.01428	0.3723	-3.25486	0.00113	down
hsa-miR-3683-5p	0.83025	0.09062	-0.00786	0.34736	-3.64437	0.00027	down
hsa-miR-6503-3p	0.8858	0.08009	-0.04277	0.27116	-4.81734	0	down
hsa-miR-6882-5p	0.7716	0.09719	0.0379	0.41889	-2.7945	0.0052	down
hsa-miR-874-3p	0.9321	0.04777	-0.02572	0.16152	-9.04583	0	down
hsa-miR-1468-5p	0.92593	0.05022	0.82751	1.02435	8.48198	0	up
hsa-miR-16-1-3p	0.82099	0.09813	-0.01332	0.37135	-3.27102	0.00107	down
hsa-miR-2277-5p	0.90123	0.06036	-0.01954	0.21707	-6.64729	0	down
hsa-miR-3163	0.80247	0.08957	0.62692	0.97802	3.37695	0.00073	up
hsa-miR-3202	0.60494	0.11742	0.16491	0.62521	-0.89366	0.3715	down
hsa-miR-382-3p	0.69136	0.10619	0.10052	0.51676	-1.80211	0.07153	down
hsa-miR-4661-5p	0.83025	0.07948	0.67447	0.98602	4.15521	3.00E-05	up
hsa-miR-6815-5p	0.77778	0.09365	0.59423	0.96133	2.96617	0.00302	up
hsa-miR-1285-5p	0.87037	0.06763	-0.00293	0.26219	-5.47622	0	down
hsa-miR-188-5p	0.81481	0.08318	0.02215	0.34822	-3.78466	0.00015	down
hsa-miR-3136-5p	0.88889	0.06166	-0.00975	0.23197	-6.3065	0	down
hsa-miR-5001-3p	0.88889	0.0657	-0.01767	0.23989	-5.91884	0	down
hsa-miR-5010-5p	0.60494	0.10961	0.39012	0.81976	0.95742	0.33836	up
hsa-miR-627-3p	0.87037	0.06763	-0.00293	0.26219	-5.47622	0	down
hsa-miR-106a-3p	0.84568	0.07663	0.00412	0.30452	-4.51089	1.00E-05	down
hsa-miR-3613-3p	0.85185	0.07263	0.0058	0.2905	-4.84442	0	down
hsa-miR-4286	0.80864	0.09919	-0.00305	0.38576	-3.11171	0.00186	down
hsa-miR-501-5p	0.87037	0.07016	-0.00788	0.26714	-5.2791	0	down
hsa-miR-584-3p	0.84568	0.07429	0.00871	0.29993	-4.65291	0	down
hsa-miR-6511b-3p	0.88272	0.06528	-0.01067	0.24524	-5.8623	0	down
hsa-miR-766-3p	0.90741	0.05672	-0.01858	0.20377	-7.18235	0	up
hsa-let-7g-3p	0.82716	0.0973	-0.01786	0.36354	-3.36251	0.00077	down
hsa-miR-101-5p	0.95679	0.03518	-0.02575	0.11217	-12.9834	0	down
hsa-miR-190b-5p	0.88272	0.06578	-0.01165	0.24622	-5.81772	0	down
hsa-miR-660-3p	0.96296	0.03168	-0.02505	0.09913	-14.614	0	down
hsa-miR-494-3p	0.80864	0.08563	0.02354	0.35918	-3.60458	0.00031	down
hsa-miR-579-5p	0.81481	0.09528	-0.00155	0.37192	-3.30421	0.00095	down
hsa-miR-1273c	0.7963	0.08631	0.03455	0.37286	-3.43308	6.00E-04	down
hsa-miR-362-3p	0.97531	0.02711	-0.02845	0.07783	-17.5299	0	down
hsa-miR-4668-5p	0.87963	0.08572	-0.04764	0.28838	-4.42872	1.00E-05	down
hsa-miR-548u-3p	0.87654	0.07057	-0.01485	0.26177	-5.33589	0	down
hsa-miR-4521	0.91358	0.05555	-0.02245	0.19529	-7.4458	0	down
hsa-miR-7-1-3p	0.82716	0.0795	0.01703	0.32865	-4.11542	4.00E-05	down

hsa-miR-550a-3p	0.93827	0.04838	-0.03309	0.15655	-9.05901	0	down
hsa-miR-580-3p	0.85185	0.07349	0.0041	0.29219	-4.78755	0	down
hsa-miR-183-3p	0.97531	0.02353	-0.02143	0.07081	-20.2007	0	down
hsa-miR-378a-5p	0.88272	0.06478	-0.00968	0.24425	-5.90791	0	down
hsa-miR-3200-5p	0.92593	0.05017	-0.02425	0.1724	-8.49048	0	down
hsa-miR-5695-3p	0.90123	0.05931	-0.01747	0.21501	-6.76537	0	down
hsa-let-7a-3p	0.74074	0.09677	0.55107	0.93041	2.48766	0.01286	down
hsa-miR-132-3p	0.82099	0.08644	0.0096	0.34842	-3.71359	2.00E-04	down
hsa-miR-3135a	0.85185	0.07505	0.00104	0.29525	-4.68793	0	down
hsa-miR-486-3p	0.95679	0.03603	0.88617	1.02741	12.67719	0	up
hsa-miR-145-5p	0.91975	0.05337	-0.02435	0.18485	-7.86508	0	down
hsa-miR-3143	0.85185	0.0889	-0.02609	0.32238	-3.95798	8.00E-05	down
hsa-miR-505-3p	0.91358	0.0528	-0.01706	0.1899	-7.83308	0	down
hsa-miR-424-5p	0.91975	0.05299	-0.02361	0.18411	-7.92139	0	down
hsa-miR-18b-5p	0.90123	0.06413	-0.02692	0.22445	-6.25676	0	down
hsa-miR-16-2-3p	0.83951	0.07737	0.68786	0.99116	4.38783	1.00E-05	up
hsa-miR-4511-5p	0.88272	0.06427	-0.00869	0.24326	-5.95461	0	down
hsa-miR-6501-5p	0.74074	0.1158	0.03229	0.48622	-2.07892	0.03762	down
hsa-miR-29b-2-5p	0.96914	0.03314	-0.03409	0.09582	-14.156	0	down
hsa-miR-26b-3p	0.89506	0.05963	-0.01194	0.22182	-6.62498	0	down
hsa-miR-1307-5p	0.89506	0.07421	-0.04052	0.25039	-5.32329	0	down
hsa-miR-190a-5p	0.88272	0.06955	-0.01903	0.2536	-5.50288	0	down
hsa-miR-624-5p	0.90123	0.05823	-0.01537	0.2129	-6.88999	0	down
hsa-miR-181a-3p	0.80247	0.09919	0.00313	0.39193	-3.04948	0.00229	down
hsa-miR-1287-5p	0.87037	0.07105	-0.00963	0.26889	-5.21276	0	down
hsa-miR-4746-5p	0.9321	0.05679	0.8208	1.0434	7.60904	0	up
hsa-let-7i-3p	0.88272	0.06636	-0.01277	0.24734	-5.76753	0	down
hsa-miR-3200-3p	0.96296	0.03144	-0.02458	0.09866	-14.7254	0	down
hsa-miR-326-3p	0.84877	0.08946	-0.02411	0.32657	-3.89853	1.00E-04	down
hsa-miR-29c-3p	0.88272	0.06987	-0.01967	0.25423	-5.47725	0	down
hsa-miR-17-3p	0.82099	0.08197	0.01835	0.33968	-3.91572	9.00E-05	down
hsa-miR-6842-3p	0.88889	0.06643	0.75869	1.01909	5.85423	0	up
hsa-miR-29c-5p	0.83333	0.0815	0.00693	0.3264	-4.0901	4.00E-05	down
hsa-miR-335-5p	0.75309	0.09426	0.06217	0.43166	-2.68498	0.00725	down
hsa-miR-532-3p	0.94444	0.04086	-0.02452	0.13563	-10.8781	0	down
hsa-miR-93-3p	0.97531	0.02364	-0.02163	0.07102	-20.1093	0	down
hsa-miR-324-3p	0.87654	0.07272	-0.01906	0.26598	-5.17834	0	down
hsa-miR-361-5p	0.79012	0.08933	0.03479	0.38497	-3.24763	0.00116	down
hsa-miR-199b-5p	0.82099	0.0844	0.01359	0.34443	-3.80321	0.00014	down
hsa-miR-362-5p	0.88889	0.06264	-0.01166	0.23388	-6.20846	0	down
hsa-miR-339-5p	0.8642	0.07293	-0.00715	0.27875	-4.9935	0	down
hsa-miR-3688-3p	0.83333	0.07831	0.01318	0.32015	-4.25657	2.00E-05	down
hsa-miR-1843	0.93827	0.04663	0.84688	1.02966	9.39933	0	up
hsa-miR-421	0.90741	0.0573	-0.01971	0.2049	-7.11028	0	down
hsa-miR-342-3p	0.94444	0.03998	-0.02281	0.13392	-11.1157	0	down
hsa-miR-324-5p	0.9321	0.04845	-0.02706	0.16286	-8.91861	0	down

hsa-miR-331-3p	0.92593	0.05515	-0.03401	0.18216	-7.72367	0	down
hsa-miR-18a-5p	0.8642	0.07311	-0.00748	0.27909	-4.9817	0	down
hsa-miR-130b-3p	0.7963	0.08729	0.03261	0.3748	-3.39424	0.00069	down
hsa-miR-3613-5p	0.78395	0.10681	0.00671	0.42539	-2.65848	0.00785	down
hsa-miR-425-3p	0.90123	0.06743	-0.03339	0.23092	-5.95082	0	down
hsa-miR-7706	0.85802	0.07961	0.70199	1.01406	4.49709	1.00E-05	up
hsa-miR-345-5p	0.93827	0.04717	-0.03071	0.15417	-9.29218	0	down
hsa-miR-29b-3p	0.87037	0.07737	-0.02201	0.28127	-4.78695	0	down
hsa-miR-126-5p	0.82716	0.10038	-0.02391	0.36959	-3.25911	0.00112	down
hsa-miR-148b-5p	0.95679	0.03394	-0.02332	0.10974	-13.4578	0	down
hsa-miR-660-5p	0.89506	0.06114	-0.01489	0.22476	-6.46207	0	down
hsa-miR-130a-3p	0.80247	0.08688	0.02725	0.36782	-3.48137	5.00E-04	down
hsa-miR-181a-2-3p	0.89506	0.06582	0.76605	1.02407	6.0019	0	up
hsa-miR-210-3p	0.8642	0.07044	-0.00225	0.27386	-5.17055	0	down
hsa-miR-503-5p	0.60494	0.13168	0.13698	0.65314	-0.79693	0.42549	down
hsa-miR-29a-3p	0.85802	0.07373	-0.00252	0.28647	-4.8562	0	down
hsa-miR-150-5p	0.85185	0.07783	-0.00439	0.30068	-4.52102	1.00E-05	down
hsa-miR-96-5p	0.87037	0.09122	-0.04916	0.30842	-4.06015	5.00E-05	down
hsa-miR-374a-5p	0.77778	0.10725	0.01201	0.43244	-2.5899	0.0096	down
hsa-miR-15a-5p	0.85185	0.07724	-0.00324	0.29954	-4.55529	1.00E-05	down
hsa-miR-454-3p	0.90123	0.07177	-0.04191	0.23944	-5.59035	0	down
hsa-miR-19b-3p	0.87654	0.07358	-0.02075	0.26766	-5.11768	0	down
hsa-miR-23a-3p-23b-3p	0.84568	0.07855	0.00037	0.30828	-4.40077	1.00E-05	down
hsa-miR-223-3p	0.8642	0.07276	-0.00681	0.27841	-5.00537	0	down
hsa-miR-30e-3p	0.94444	0.04418	0.85786	1.03103	10.06028	0	down
hsa-miR-576-3p	0.78395	0.08955	0.04053	0.39156	-3.17086	0.00152	down
hsa-miR-20b-5p	0.83333	0.0861	-0.00209	0.33542	-3.8714	0.00011	down
hsa-miR-142-3p	0.81481	0.10215	-0.01502	0.38539	-3.08193	0.00206	down
hsa-miR-128-3p	0.91975	0.05203	0.81778	1.02173	8.06761	0	up
hsa-miR-652-3p	0.87654	0.08649	-0.04607	0.29298	-4.35339	1.00E-05	down
hsa-miR-106b-5p	0.84568	0.10743	-0.05624	0.36488	-3.21774	0.00129	down
hsa-miR-15b-5p	0.83951	0.10138	-0.03821	0.3592	-3.34874	0.00081	down
hsa-miR-146a-5p	0.7963	0.09546	0.0166	0.39081	-3.10381	0.00191	down
hsa-miR-425-5p	0.88272	0.10466	-0.08785	0.32242	-3.65665	0.00026	down
hsa-miR-144-3p	0.87654	0.09361	-0.06001	0.30692	-4.02261	6.00E-05	down
hsa-miR-181a-5p	0.77778	0.11597	-0.00508	0.44953	-2.39516	0.01661	down
hsa-miR-20a-5p	0.76543	0.11531	0.00857	0.46057	-2.30193	0.02134	down
hsa-miR-106a-5p-17-5p	0.82716	0.09776	-0.01877	0.36445	-3.34651	0.00082	down
hsa-miR-30e-5p	0.83951	0.11598	-0.06682	0.3878	-2.92735	0.00342	down
hsa-miR-93-5p	0.85185	0.10569	-0.059	0.3553	-3.32906	0.00087	down
hsa-miR-16-5p	0.82716	0.11233	-0.04733	0.39301	-2.91243	0.00359	down
hsa-miR-151a-3p	1	0	1	1	Inf	0	up
hsa-miR-191-5p	0.76543	0.11574	0.00771	0.46142	-2.29325	0.02183	down
hsa-miR-103a-3p-107	0.78395	0.1097	0.00105	0.43105	-2.58849	0.00964	down
hsa-let-7a-5p-7c-5p	0.88272	0.07596	0.73384	1.0316	5.03838	0	up

hsa-miR-148a-3p	1	0	1	1	Inf	0	up
hsa-miR-92a-3p	0.8642	0.07083	0.72537	1.00302	5.14188	0	up
hsa-miR-486-5p	0.7716	0.09341	0.58853	0.95468	2.9077	0.00364	up
hsa-miR-625-5p	0.60185	0.14347	0.32066	0.88305	0.70992	0.47776	down
hsa-miR-937-3p	0.81746	0.08456	0.65172	0.9832	3.7541	0.00017	up
hsa-miR-2355-3p	0.88889	0.06821	-0.02258	0.2448	-5.70142	0	down

Supplementary Table S3: Table shows the statistics of differentially expressed miRNAs based on volcano analysis. The statistics include the area under the curve (AUC) value and its standard error (SE), confidence interval (Upper and Lower Limits) and statistical significance (z score and p-value). The standard error and confidence interval were calculated using DeLong method.
