# Supplementary material for: Interplay Between Dysregulated Immune System and the Footprints of Blood-Borne miRNAs in Treatment Naive Crohn’s Disease and Ulcerative Colitis Patients
Source: Int J Mol Sci. 2025 Dec 15;26(24):12042. doi: 10.3390/ijms262412042 (PMC12732772; doi:10.3390/ijms262412042)
Supplement: Supplementary file 1 [file ijms-26-12042-s001.zip › Supplementary_Table S4.pdf]

microRNA	Sequence (5′-3′)	Assay ID
hsa-let-7i-5p*	UGAGGUAGUAGUUUGUGCUGUU	478375_mir
hsa-miR-664b-3p	UUCAUUUGCCUCCCAGCCUACA	479148_mir
hsa-miR-23c	AUCACAUUGCCAGUGAUUACCC	478783_mir
hsa-miR-874-3p	CUGCCCUGGCCCGAGGGACCGA	478205_mir
hsa-miR-1468-5p	CUCCGUUUGCCUGUUUCGCUG	479578_mir
hsa-miR-2277-5p	AGCGCGGGCUGAGCGCUGCCAGUC	479595_mir
hsa-miR-3163	UAUAAAAUGAGGGCAGUAAGAC	479648_mir
hsa-miR-766-3p	ACUCCAGCCCCACAGCCUCAGC	478342_mir
hsa-miR-101-5p	CAGUUAUCACAGUGCUGAUGCU	478620_mir
hsa-miR-660-3p	ACCUCCUGUGUGCAUGGAUUA	479143_mir
hsa-miR-362-3p	AACACACCUAUUCAAGGAUUCA	478058_mir
hsa-miR-4521	GCUAAGGAAGUCCUGUGCUCAG	478916_mir
hsa-miR-550a-3p	UGUCUUACUCCCUCAGGCACAU	479032_mir
hsa-miR-183-3p	GUGAAUUACCGAAGGGCCAUAA	477936_mir
hsa-miR-3200-5p	AAUCUGAGAAGGCGCACAAGGU	478021_mir
hsa-miR-5695-3p	ACUCCAAGAAGAAUCUAGACAG	480154_mir
hsa-miR-486-3p	CGGGGCAGCUCAGUACAGGAU	478422_mir
hsa-miR-145-5p	GUCCAGUUUUCCCAGGAAUCCCU	477916_mir
hsa-miR-505-3p	CGUCAACACUUGCUGGUUUCCU	478145_mir
hsa-miR-424-5p	CAGCAGCAAUUCAUGUUUUGAA	478092_mir
hsa-miR-18b-5p	UAAGGUGCAUCUAGUGCAGUUAG	478584_mir
hsa-miR-16-2-3p	CCAAUAUUACUGUGCUGCUUUA	477931_mir
hsa-miR-29b-2-5p	CUGGUUUCACAUGGUGGCUUAG	478003_mir
hsa-miR-624-5p	UAGUACCAGUACCUUGUGUUCA	478178_mir
hsa-miR-4746-5p	CCGGUCCCAGGAGAACCUGCAGA	479999_mir
hsa-miR-3200-3p	CACCUUGCGCUACUCAGGUCUG	478322_mir
hsa-miR-17-3p	ACUGCAGUGAAGGCACUUGUAG	477932_mir
hsa-miR-532-3p	CCUCCCACACCCAAGGCUUGCA	478336_mir
hsa-miR-93-3p	ACUGCUGAGCUAGCACUUCCCG	478209_mir
hsa-miR-1843	UAUGGAGGUCUCUGUCUGGC	483104_mir
hsa-miR-421	AUCAACAGACAUUAAUUGGGCGC	478088_mir
hsa-miR-342-3p	UCUCACACAGAAAUCGCACCCGU	478043_mir
hsa-miR-324-5p	CGCAUCCCCUAGGGCAUUGGUG	483066_mir
hsa-miR-331-3p	GCCCCUGGGCCUAUCCUAGAA	478323_mir
hsa-miR-425-3p	AUCGGGAAUGUCGUGUCCGCCC	478093_mir
hsa-miR-345-5p	GCUGACUCCUAGUCCAGGGCUC	478366_mir
hsa-miR-29b-3p	UAGCACCAUUUGAAAUCAGUGUU	478369_mir
hsa-miR-126-5p	CAUUAUUACUUUUGGUACGCG	477888_mir
hsa-miR-148b-5p	AAGUUCUGUUAUACACUCAGGC	478719_mir
hsa-miR-29a-3p	UAGCACCAUCUGAAAUCGGUUA	478587_mir
hsa-miR-374a-5p	UUAUAAUACAACCUGAUAAGUG	478238_mir
hsa-miR-15a-5p	UAGCAGCACAUAAUGGUUUGUG	477858_mir
hsa-miR-20b-5p	CAAAGUGCUCAUAGUGCAGGUAG	477804_mir

hsa-miR-19b-3p	UGUGCAAAUCCAUGCAAAACUGA	478264_mir
hsa-miR-30e-3p	CUUUCAGUCGGAUGUUUACAGC	478388_mir
hsa-miR-128-3p	UCACAGUGAACCGGUCUCUUU	477892_mir
hsa-miR-652-3p	AAUGGCGCCACUAGGGUUGUG	478189_mir
hsa-miR-106b-5p	UAAAGUGCUGACAGUGCAGAU	478412_mir
hsa-miR-15b-5p	UAGCAGCACAUCAUGGUUUACA	478313_mir
hsa-miR-146a-5p	UGAGAACUGAAUUCCAUGGGUU	478399_mir
hsa-miR-425-5p	AAUGACACGAUCACUCCCGUUGA	478094_mir
hsa-miR-144-3p	UACAGUAUAGAUGAUGUACU	477913_mir
hsa-miR-20a-5p	UAAAGUGCUUAUAGUGCAGGUAG	478586_mir
hsa-miR-106a-5p	AAAAGUGCUUACAGUGCAGGUAG	478225_mir
hsa-miR-30e-5p	UGUAAACAUCCUUGACUGGAAG	479235_mir
hsa-miR-93-5p	CAAAGUGCUGUUCGUGCAGGUAG	478210_mir
hsa-miR-16-5p	UAGCAGCACGUAAAUAUUGGCG	477860_mir
hsa-miR-151a-3p	CUAGACUGAAGCUCCUUGAGG	477919_mir
hsa-miR-191-5p	CAACGGAAUCCCAAAAGCAGCUG	477952_mir
hsa-miR-103a-3p	AGCAGCAUUGUACAGGGCUAUGA	478253_mir
hsa-miR-148a-3p	UCAGUGCACUACAGAACUUUGU	477814_mir
hsa-miR-92a-3p	UAUUGCACUUGUCCCGGCCUGU	477827_mir
hsa-miR-486-5p	UCCUGUACUGAGCUGCCCCGAG	478128_mir

Supplementary Table S4. MicroRNA primer sequences used in RT-qPCR reactions (Thermo Fisher Scientific, USA). *endogenous control
