# Supplementary material for: Interplay Between Dysregulated Immune System and the Footprints of Blood-Borne miRNAs in Treatment Naive Crohn’s Disease and Ulcerative Colitis Patients
Source: Int J Mol Sci. 2025 Dec 15;26(24):12042. doi: 10.3390/ijms262412042 (PMC12732772; doi:10.3390/ijms262412042)
Supplement: Supplementary file 1 [file ijms-26-12042-s001.zip › Supplementary_Table S5.pdf]

Sample_name	hsa-miR-145-5P	hsa-miR-101-3p	hsa-miR-106b-5p	hsa-miR-15a-5p	hsa-miR-17-3p	hsa-miR-151a-3p	hsa-miR-103a-3p	hsa-miR-16-5p	hsa-miR191-5P	hsa-miR-19b-3p	hsa-miR-20a-5p	hsa-miR-103a-3p	hsa-miR-16-2-3p	hsa-miR-106a-5p
CD1	31.11	23.37	22.61	20.2	20.99	25.8	24.52	18.28	22	22.36	21.82	24.52	19.98	22.87
CD2	32.73	22.8	22.56	27.65	20.98	27.65	24.36	19.98	22.01	22.52	21.83	24.36	18.85	22.81
CD3	32.74	25.17	22.585	25.56	20.83	25.56	24.44	19.95	23.46	22.44	22.11	24.44	24.91	22.84
CD4	30.09	22.3	26.48	26.54	20.52	26.54	24.49	18.85	22.42	21.91	22.1	24.61	28.55	22.14
CD5	26.57	20.62	27.86	19.77	19.53	26.42	24.49	18.84	25.03	21.69	22.105	24.09	24.1	22.11
CD6	26.01	20.39	26.29	21.7	18.42	24.75	24.5	19.61	20.93	22.56	22.7	24.03	23.52	22.14
CD7	26.57	19.39	25.18	19.08	16.69	23.57	24.81	19.78	22.59	22.78	22.62	24.54	25.38	22.18
CD8	25.95	19	24.74	18.04	16.73	22.23	24.77	19.92	22.88	22.69	22.53	24.26	23.2	22.74
CU1	28.8	21.93	21.31	17.83	19.92	24.69	22.94	17.09	22.48	21.4	20.55	22.94	20.51	23.98
CU2	29.9	20.22	21.18	15.15	18.87	27.57	22.98	20.51	22.49	21.32	20.54	22.98	19.51	24.11
CU3	28.18	19.18	24.14	16.01	18	27.27	22.96	20.38	22.5	21.36	20.545	22.96	21.79	24.045
CU4	28.05	26.93	24.21	27.57	18.23	28.37	22.56	19.51	21.91	21.18	21.825	22.7	22.58	23.73
CU5	29.14	20.38	23.52	27.27	17.62	24.29	22.59	21.79	21.69	20.93	20.52	22.62	22.18	24
CU6	26.79	20.01	22.99	28.37	16.72	23.06	22.6	21.93	21.81	21.055	20.69	22.53	28.92	23.11
CU7	26.13	19.17	22.9	17.17	16.83	26.39	22.59	21.74	21.79	21.18	20.76	22.16	22.9	23.09
CU8	26.79	19.57	23.6	17.23	18.23	24.39	23.2	21.86	22.28	20.93	20.81	22.07	22.77	28.98
CU9	27.24	19.29	23.33	17.94	17.18	23.78	23.16	23.96	21.79	20.93	20.42	22.15	23.08	28.93
CU10	25.82	19.16	21.02	17.49	18.09	23.05	23.73	23.73	21.07	21.055	20.37	22.62	22.79	23.28
control1	31.12	21.77	20.99	16.84	19.64	22.58	24	17	22.13	20.63	20.28	22.53	21.09	23.2
control2	30.24	21.81	21.005	16.15	19.66	25.04	22.53	17.01	21.76	20.7	20.27	22.55	25.61	23.24
control3	29.96	21.79	24.75	18.72	19.65	25.08	22.55	17	21.27	20.665	20.275	22.54	25.58	23.55
control4	30.84	21.99	24.61	18.76	19.12	25.06	22.54	17.78	22	20.99	20.19	22.83	25.595	23.56
control5	30.83	21.85	24.54	18.74	18.86	25.8	22.19	17.96	22.01	20.93	20.22	22.81	18.41	23.2
control6	27.3	21.91	24.79	18.72	18.63	25.81	22.18	18	22.005	18.3	20.14	22.82	18.51	23.24
control7	27.04	21.69	24.64	18.76	18.09	25.805	23.02	17.56	22.99	19.08	20.26	22.14	18.35	21.07
control8	26.93	22.2	24.32	18.74	17.96	26.17	23.05	17.63	22.59	19.36	20.56	22.08	25.29	23.21
control9	27.3	22.3	24.14	18.72	17.6	26.16	24.77	18.02	22.38	19.76	20.46	22.45	25.16	23.215

Supplementary Table S5: Raw Ct values of RT-qPCR validation of miRNAs per sample. CD (Crohn's disease), UC (Ulcerative colitis)
